# Development of quality assessment tool for systematic reviews and meta-analyses of real-world studies: a Delphi consensus survey

**DOI:** 10.1007/s00296-024-05595-4

**Published:** 2024-04-29

**Authors:** Tadesse Gebrye, Chidozie Mbada, Zalmai Hakimi, Francis Fatoye

**Affiliations:** 1 Department of Health Professions, Faculty of Health, Psychology, and Social Care, Manchester Metropolitan University, Brooks Building, Birley Fields Campus, 53 Bonsall Street, Manchester, M15 6GX UK; 2 Lifestyle Diseases, Faculty of Health Sciences, North-West University, Potchefstroom, South Africa; 3 Sobi AB, Stockholm, Sweden

**Keywords:** Quality assessment tool, Delphi technique, Real-world, Survey

## Abstract

The increasing adoption of real-world studies in healthcare for decision making and planning has further necessitated the need for a specific quality assessment tool for evidence synthesis. This study aimed to develop a quality assessment tool for systematic reviews (SR) and meta-analysis (MA) involving real-world studies (QATSM-RWS) using a formal consensus method. Based on scoping review, the authors identified a list of items for possible inclusion in the quality assessment tool. A Delphi survey was formulated based on the identified items. A total of 89 experts, purposively recruited, with research experience in real-world data were invited to participate in the first round of Delphi survey. The participants who responded in the first Delphi round were invited to participate (n = 15) in the phrasing of the items. Strong level of agreement was found on the proposed list of items after the first round of Delphi. A rate of agreement ≥ 0.70 was used to define which items to keep in the tool. A list of 14 items emerged as suitable for QATSM-RWS. The items were structured under five domains: introduction, methods, results, discussions, and others. All participants agreed with the proposed phrasing of the items. This is the first study that has developed a specific tool that can be used to appraise the quality of SR and MA involving real-world studies. QATSM-RWS may be used by policymakers, clinicians, and practitioners when evaluating and generating real-world evidence. This tool is now undergoing validation process.

## Introduction

The role of real-world studies in regulatory, drug development, and healthcare is becoming increasingly important for decision making and planning [1]. Real-world studies are studies generated from real-world data which are related to patient health status or delivery of health care routinely collected from a variety of sources including the internet, social media, wearable devices, and mobile devices, claims and billing activities, registries, electronic health records (EHRs), product and disease registries, and e-health services [2]. The rising costs of traditional clinical trials and the fast development of artificial intelligence and machine learning techniques have prompted the interest in the use of real-world data [3, 4]. Real-world studies help provide an overall picture of safety of any medical product (adverse effects) that randomised control trials by themselves do not provide [5]. Real-world studies support randomised control trials, in the case of rare diseases, when recruiting suitable patients is difficult [6].

The aim of conducting real-world studies is to improve healthcare delivery and health outcomes for patients. Systematic reviews and clinical guidelines of these type of studies bring together trustworthy information and transferring this information into clinical, management, and policy arenas [7]. Several quality assessment tools have previously been developed to improve the quality of reporting of systematic reviews and meta-analysis of studies based on randomised trials and observational studies [8–13]. These quality assessment tools are useful to evaluate studies with a non-biased approach for a given topic [14]. When reading any type of evidence, being critical of all aspects of the study design, execution and reporting is vital before being applied to practice.

Increasing research studies and the precise nature of scientific discoveries may highlight the importance of developing quality assessment tools. The quality checklist of a given assessment tools may not always suit as included items may not be relevant for the purposes of the intended SR and MA [15, 16]. Thus, individual real- worlds studies included in SRs and MA should be assessed in terms of quality of reporting or potential risk of bias using its own specific assessment tool [17]. A formal quality assessment of the individual studies included in SRs and MA of real-world studies is important to evaluate the overall quality of their results. A scoping review on quality assessment tools used in SRs and MA of real-world studies identified no validated and reliable tool that are specific to these types of study [18].

Due to their unique design features, the criteria needed to assess the quality of real-world studies may differ from other studies. The adoption of a standardised QATSM-RWS may also avoid the use of different types of tools that could be a source of bias. Although several types of quality assessment tools are currently used [18], none of these have been systematically developed to evaluate SRs and MA involving real-world studies. This study was aimed to develop QATSM-RWS using a formal consensus method.

## Methods

### Study design

This study was approved by the Health and Education Research Ethics and Governance Committee at The Manchester Metropolitan University (EthOS Reference Number: 56368). From an initial scoping review [18], the authors identified 16 quality assessment tools that were used to assess the quality of systematic reviews and meta-analyses involving real-world studies. Using Excel spreadsheet, the list of items used by the 16 quality assessment tools were listed and helped to develop themes. Those items used as a criteria of quality assessment by more than 50% of the included studies were selected for inclusion in the proposed quality assessment tool (Table 1). The authors were responsible for item generation of the initial checklist, whereas a Delphi group of experts adapted and approved the final checklist. Consensus on eligible professionals was based on track-record and evidence of publication of real-world studies. To minimise bias, all researchers were blinded to the experts’ identities that participated to the Delphi survey. All questionnaires were developed and facilitated using the online survey platform.

**Table 1:** Ranked list of items of the quality assessment tool

List of items	Rank (median)	Mean (SD)	% of Agreement
Inclusion of research questions/objectives	4 (4)	4 (0.00)	100
Inclusion of the scientific background and rationale for the investigation being reported	4 (4)	3.9 (0.35)	86.7
Study sample description and definition	4 (4)	3.9 (0.52)	93.3
Description of the data sources	4 (4)	3.9 (0.35)	86.7
Description of study design and data analysis	4 (4)	3.6 (0.83)	86.7
Inclusion of adequate sample size	4 (4)	3.9 (0.35)	73.3
Description of inclusion and exclusion criteria	4 (4)	3.8 (0.41)	80.0
Description and appropriate choice of end point for the study	4 (4)	3.8 (0.41)	80.0
Description of appropriate follow-up period or last update to the major endpoints	4 (4)	3.9 (0.35)	86.7
Description of sufficient methods to enable them to be repeated	4 (4)	3.9 (0.35)	86.7
Description of key findings	4 (4)	3.9 (0.35)	86.7
Justification of the discussions and conclusions by the key findings of the study	4 (4)	3.8 (0.41)	80.0
Inclusion of potential conflict of interest of study researcher(s) and funder(s)	4 (4)	3.7 (0.46)	73.3
Inclusion of any funding sources that may affect the authors' interpretation of the results	4 (4)	3.7 (0.46)	73.3

The Delphi method was used to achieve expert consensus on the content of QATSM-RWS. Delphi method is useful to obtain consensus on an important topic using a structured multi-staged survey involving professionals in the area [19].

### Participants

There are no universally agreed criteria for the selection of experts for a Delphi study [20]. The selection of participants was professionals from various locations. One of the authors (TG) identified participants who published studies that have used real-world data. Participants were included if they hold a postgraduate qualification, recognised through publication of real-world studies, and taught at university level. On the other hand, professionals who did not have expertise in real-world studies were excluded. Informed consent was obtained from each participant at the start of the survey, by providing participant information and requesting that participants indicate consent by clicking on the consent box. Participation was voluntary and was kept confidential throughout the study.

### Study procedures

We employed a purposive sampling strategy. An invitation email was sent to the individuals who published studies using real-world data informing them of the purpose and details of the study. Their emails were obtained from the contact details of the published article. Agreeing to participate entailed clicking on the survey link to initiate the first round. The Delphi group size depends more on group dynamics in reaching consensus among experts, we aimed for the recommended minimum sample size of 15 [21]. To form a representative international expert, we included professionals from various countries.

In Delphi survey round one, the participants (n = 89) were asked to rate each item on a four-point rating scale (1 = strongly disagree, 2 = disagree, 3 = agree, 4 = strongly agree). Comment boxes were provided, allowing the participants to provide recommendations regarding any additions and/or deletions to the list of proposed items. Each survey required not more than 10 min to complete. For a Delphi study, a universally agreed minimum level of consensus does not exist but typically ranges from 50 to 80% [22]. In this study, consensus was defined a priori to include items that had a mean score of ≥ 3.5 and were rated agreed (3) or strongly agreed (4) or by ≥ 70% of the participants. Items that did not meet an agreement of ‘agreed’ or ‘strongly agreed’ for at least 70% of the participants were considered for the next round.

**Fig. 1:**
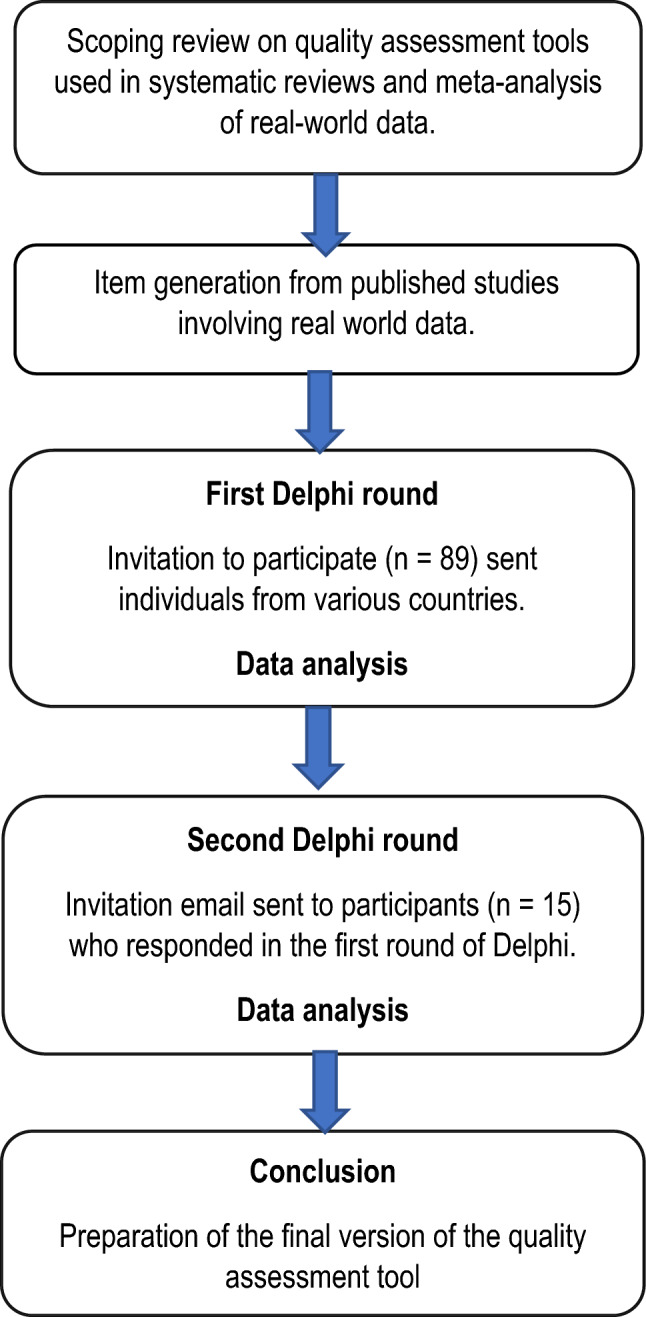
Flowchart of the Delphi process

In round two, the participants (n = 15) who responded in the first Delphi round were invited to participate. The findings of the first round were reported to the participants and were asked to ‘agree’, ‘’not agree’’ with the proposed phrasing of the included items. If the participants did not agree with the phrasing, they were asked to suggest alternative phrasing or comments rather than ranking the question like the first round. The flowchart of the Delphi process is shown in Fig. 1.

### Statistical analysis

Descriptive analysis was performed by calculating the mean scores, standard deviation (SD) and percentage for each item. All data analysis was done using the statistical software SPSS Statistics version 24.0 (IBM). Greater importance was associated with higher means and lower SDs.

## Results

**Fig. 2:**
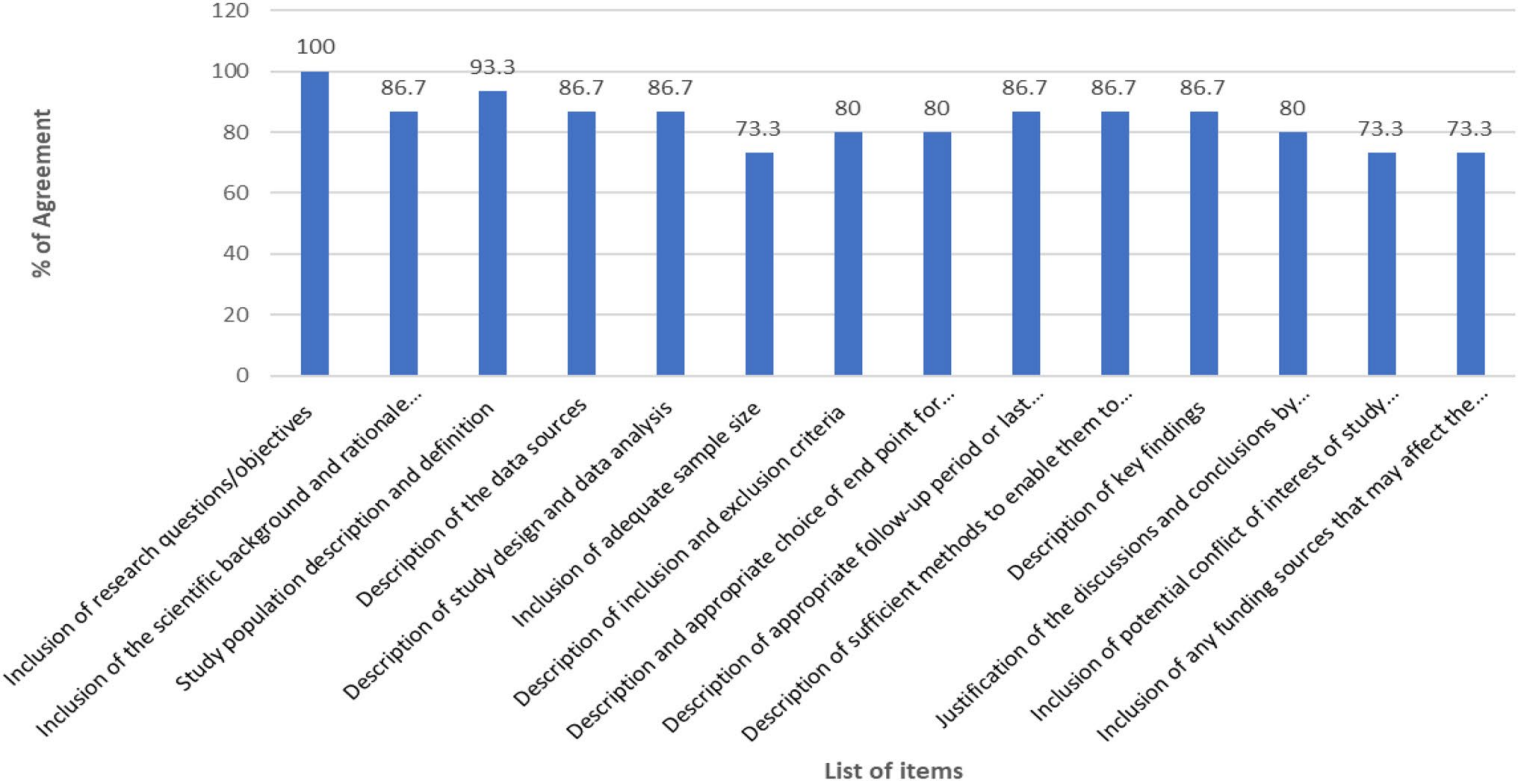
A visual representation of the % of agreement of the list of items of the quality assessment tool

The items/domains used for quality assessment by most of published SR and MA involving real-world data were considered in this study. Following the scoping review, the authors developed a survey questionnaire that consisting of 14 questions. Based on the consensus criteria, all the included items have reached a sufficiently high level of consensus amongst all the respondents and were included in the quality assessment tool (Table 1 or Fig. 2). High levels of consensus were demonstrated, with one item: inclusion of research questions/objectives reaching 100% agreement of ‘strongly agreed’. The remaining items reaching ≥ 70% consensus agreement by the participants (n = 15).

In Delphi round 2, out of the 15 professionals who participated in round 1 Delphi survey, only 12 of them responded. All these participants agreed with the proposed phrasing of the included items (Table 2). However, some comments were proposed regarding the phrasing of the items. The authors considered all the comments from the participants carefully and made relevant changes. Thus, the final tool had face and content validity as judged by the consensus panel.

**Table 2:** List of items included in the QATSM-RWS and risk of bias judgment

SN	Item	Yes (1)	No (0)	Unclear (0.5)
	Introduction			
1	Was (were) the research question/objective (s) of the study clearly defined?			
2	Does the study explain the scientific background and rationale for the investigation being reported?			
	Methods			
3	Are the study sample demographic characteristics clearly described and defined?			
4	Were the sources of data used for the study clearly described?			
5	Are the study design and data analysis applied in the study described in enough detail?			
6	Was the chosen sample size appropriate the objective of the study?			
7	Are the inclusion and exclusion criteria applied to the study described in enough detail?			
8	Were the outcomes assessed in the study appropriate and clearly defined?			
9	Was the follow up of participants complete and long enough?			
10	Were the methods of the study clearly described to enable them to be repeated?			
	Results			
11	Are the reported results clear and comprehensible?			
	Discussion			
12	Were the conclusions/recommendations of the study justified and based on the study results?			
13	Was there a statement disclosing the potential conflict of interest of researcher(s)?			
	Others			
14	Was there a statement disclosing the source of funding for the study that may affect the authors' interpretation of the results?			

## Discussion

This study aimed to develop QATSM-RWS using a formal consensus method. To our knowledge, this is the first study to report QATSM-RWS developed through the Delphi method. Analysis of real-world data are needed to generate real-world evidence [23]. According to Framework for FDA's Real-World Evidence Program [24], real-world evidence is the clinical evidence about the usage and potential benefits or risks of a medical product [25–29]. However, concern relating to poor data quality due to unstructured data and resulting bias have been highlighted in using real-world data to generate evidence [25–27]. In addition, using non-specific tools to appraise quality from real-world data may further complicate evidence generated, hence the need for a specific tool for the purpose. This study adopted the Delphi approach, accordingly 15 authors in the field of real-world studies across various backgrounds and nationalities participated in the development of the tool. The main purposes of using experts in Delphi is to increase the qualitative strength of consensus [28]. Although, there is agreement in literature on the sample size required for the panel, a range between 10–100 are common in literature. As it is the case in this study, having appropriate panel size of experts who are knowledgeable in the topic area, with varied practice specialties, academic and geographical backgrounds in the process improves the generalizability of Delphi results [28, 29]. The findings of the current study revealed a high level of consensus during the first and second round among the participants about the list of items and phrasing of the quality assessment tool.

The findings of a preliminary scoping review by Gebrye et al. [18] suggested that the generic quality assessment tools are mostly used in systematic reviews and meta-analysis involving real-world studies, while no validated and reliable specific tool currently exist. A related quality assessment tool for real-world studies by Wylde et al. [30] seems to suggest that each individual item rating is reported, rather than an overall score and it is non-summative. Hence, a Delphi survey was conducted following rigorous methodical process to develop a specific quality assessment tool with face and content validity, which also has both items and an overall quality scores. Overall quality scores is the summation of each items from a quality assessment tool [17]. Despite debates regarding weighting of individual items to provide an overall quality score [17], it continues to be adopted and used as part of the quality assessment process in systematic reviews [31, 32].

Real-world data is important to produce real-world evidence that helps to answer questions that may not be addressed by clinical trials and lab-based experiments [2]. To improve the quality and transparency of evidence it is necessary that real-world evidence studies are clearly planned, conducted, and reported. Real-world evidence has the potential to significantly improve the efficiency of health-related research and decision making. Parallel to this, systematic reviews have become the standard approach in assessing and summarizing health-related research [33]. In order to generate unbiased results from systematic reviews it is important that the quality of the included studies is assessed. The authors strongly believe that the quality assessment tool developed in the current study could be used to ensure a more focussed and coordinated approach to research in the area.

This study has some strengths and limitations that needs careful consideration during its applications. The strength of this study was the spread of experts across different continents. The authors are comfortable with the rigorous methodology used to the Delphi study making it a key strength of the current study. On the other hand, the findings reported here should be viewed in the context of the limitations of this study. This quality assessment tool was developed based on expert consensus without empirical evidence for potential sources of bias in systematic reviews and meta-analysis involving real-world studies. This study used email contacts, and some authors in real-world data might have been excluded from taking part in the study. Thus, for completeness, the developed tool needs further testing in real-world studies to ensure its applicability in a realistic setting [34].

## Conclusion

A specific tool (QATSM-RWS) that can be used for quality assessment of SR and MA involving real-world studies was developed. QATSM-RWS appears to be detailed and it is easy to use. The significance of the current study is that the newly developed QATSM-RWS has implications for research, education, and practice as it may help facilitate decision making to improve access to healthcare interventions to enhance health outcomes for patients. Thus, QATSM-RWS may be used by policymakers, clinicians, and practitioners when evaluating and generating real-world evidence. This tool is now undergoing validation process.

## Data Availability

Data are available upon reasonable request from the authors.
